# Systematic Review of the Economic Evaluation of Returning Incidental Findings in Genomic Research

**DOI:** 10.3389/fpubh.2021.697381

**Published:** 2021-07-01

**Authors:** Mayara Fontes Marx, John E. Ataguba, Jantina de Vries, Ambroise Wonkam

**Affiliations:** ^1^Department of Pathology, Division of Human Genetics, Faculty of Health Sciences, University of Cape Town, Cape Town, South Africa; ^2^Health Economics Unit, School of Public Health and Family Medicine, Faculty of Health Sciences, University of Cape Town, Cape Town, South Africa; ^3^Institute to Infectious Disease and Molecular Sciences, Faculty of Health Sciences, University of Cape Town, Cape Town, South Africa

**Keywords:** economic evaluation, genetic research, systematic review, incidental (secondary) findings, costs

## Abstract

**Objectives:** Discussions regarding who and how incidental findings (IFs) should be returned and the ethics behind returning IFs have increased dramatically over the years. However, information on the cost and benefits of returning IFs to patients remains scanty.

**Design:** This study systematically reviews the economic evaluation of returning IFs in genomic sequencing. We searched for published articles on the cost-effectiveness, cost-benefit, and cost-utility of IFs in Medline, Scopus, PubMed, and Google Scholar.

**Results:** We found six published articles that met the eligibility criteria of this study. Two articles used cost analysis only, one used cost-benefit analysis only, two used both cost analysis and cost-effectiveness, and one used both cost-benefit analysis and cost-utility to describe the cost of returning IFs in genomic sequencing.

**Conclusion:** While individuals value the IF results and are willing to pay for them, the cost of returning IFs depends on the primary health condition of the patient. Although patients were willing to pay, there was no clear evidence that returning IFs might be cost-effective. More rigorous economic evaluation studies of IFs are needed to determine whether or not the cost of returning IFs is beneficial to the patient.

## Introduction

Genomic sequencing tests are used in clinical settings to identify the genetic cause of illness or the likelihood of an individual to develop a particular health condition (1, 2), and they are commonly used in research as well. In some instances, when using whole-genome sequencing (WGS) or whole-exome sequencing (WES), incidental or secondary findings, defined as accidental genetic results that are unrelated to the primary purpose for the test, might be uncovered (3). The American College of Medical Genetics and Genomics (ACMG) recommends that 59 actionable genes that are associated with 24 diseases should be returned to the patients (4), and similar lists have been proposed for research-related initiatives (5). These lists contain variants in pathogenic genes that are considered medically actionable and could potentially lead to improved management or preventative measures for an individual regarding a particular condition or disease.

While there is a rich discussion in the literature on how and who should return incidental findings (IFs) to patients and the ethics behind delivering the results (6, 7), however, less so in resource-constrained settings such as Africa (8, 9), little is known about the actual cost and benefits of returning the IFs to patients. There is a concern that the cost of returning those results can potentially increase health care expenditure and financial burden of patients mainly from a cascade of additional costs from, for example, follow-up visits and additional tests, which may provide very limited clinical benefits (10–12). Langanke and Erdmann (11) suggest that individuals might undertake confirmatory tests or follow-up screenings, treatments, and lifestyle changes even when they may eventually not develop the adverse health condition. In many low- and middle-income countries where households predominantly bear the cost of health services through out-of-pocket payments, additional costs from returning IFs could be impoverishing (13).

Drummond et al. (14) list four types of economic evaluation techniques (cost analysis, cost-effectiveness, cost-utility, and cost-benefit) to assess the cost and/or effects of health services, care and treatment options. The choice of a technique for empirical assessment depends, among other things, on the main objective of the evaluation. For instance, in simplistic terms, cost-effectiveness analysis assesses the cost of gaining health outcomes, e.g., life-years gained between alternative programs; cost-utility analysis considers individuals or societies preference related to a health outcome, e.g., quality-adjusted life-years (QALYs); cost-benefit analysis translates health outcomes such as life-years gained and quality of life into monetary (e.g., dollar) terms to compare programs costs; while cost analysis sums up the cost (e.g., dollar) of the program or interventions. This study only considers costs but not the effects of the alternatives being compared.

Economic evaluation is beneficial for efficient, and sometimes, equitable health care decisions as resources for health are scarce with significant budget constraints and competing demands for the available resources, especially in the public sector. As the updated ACMG guidelines suggest that patients may now opt out from receiving IFs results (12), it is crucial to assess the potential costs associated with returning IFs to individuals using economic evaluation techniques. This will provide, among other things, evidence on the benefits, preferences, and monetary costs of returning IFs to provide scientific evidence on the relative costs and benefits associated with both courses of action. Further, patients can decide on the information that they need to be returned to them. The adverse costs of events caused by returning IFs to individuals may be compared with the costs of not returning IFs.

To our knowledge, apart from the first attempt to review the cost-effectiveness of clinically actionable findings with WGS (15), this review represents the first systematic analysis of the economic evaluation of returning IFs in genomic sequencing. Douglas et al. (15) only considered the cost-effectiveness [e.g., incremental cost-effectiveness ratios (ICER)] of IFs in WGS. This review builds on the review of Douglas et al. (15), including published articles that used any economic evaluation technique to provide evidence on the cost and effects of IFs in any genomic sequencing, such as WGS, WES, and next-generation sequencing. It adds to the genomic discussion on the cost/benefits of pursuing unexpected genomic findings. Furthermore, the review will generate evidence to support the ACMG recommendation of returning IFs to individuals.

## Materials and Methods

### Search Strategy

We focused on any economic evaluation technique that provided cost evidence of returning IFs in any genomic sequencing. The literature review used subject-specific databases, such as Medline through Scopus, PubMed, and Google Scholar as of April 8, 2020 for articles using the search keywords “cost-benefit,” “cost benefit,” “economic evaluation,” “economic,” “cost^*^,” “cost utility,” “cost effectiveness,” “cost-effectiveness,” “cost analysis,” “incidental finding,” “incidental^*^,” “secondary finding^*^,” “returnable result^*^,” “unexpected findings,” “genome,” “genomics,” “ACMG,” “whole exome sequencing,” “whole genome sequencing,” “WES,” “WGS” combined by the Boolean commands “AND/OR.” The keywords were drawn from the Medical Subject Headings (MeSH terms) and from articles on ACMG recommendations for reporting secondary findings (4, 16). Additional articles were identified through reference-list searching.

### Inclusion and Exclusion Criteria

The original conception of this review was to explore the economic evaluation of returning IFs in genomic research in Africa. However, an initial review using “Africa^*^” as a geographic region filter together with the search keywords mentioned above returned only two articles (one from PubMed and one from Medline), neither of which contained a detailed statistical analysis of the outcomes variable, the economic evaluation of returning Ifs, and could not be included in this review. In response to this gap, the scope of the geographic region was broadened beyond Africa.

The following eligibility criteria were applied for studies to be included in the review: (a) only peer-reviewed articles published in English and (b) the analysis of all articles included in the review should be an economic evaluation [any of the four types of economic evaluation techniques, cost analysis, cost-effectiveness, cost-utility, and cost-benefit (14)] of returning IFs. The review excluded research protocols and commentaries articles, where IFs were not the main topic, and systematic reviews. All articles meeting the inclusion criteria and published until April 8, 2020 were included in the review.

### Study Selection

MFM and JEA first screened the titles and the abstracts according to the study inclusion and exclusion criteria. MFM and JEA, individually, extracted studies that met the inclusion criteria using Excel forms, including details on the study design, the study population, the outcome measures, and the study quality. AW checked the extracted data. Disagreements were resolved by discussion among MFM, JEA, and AW. If a decision was not reached, a fourth author (JdV) was consulted.

### Quality Assessment

The quality of studies included in the review was assessed using the quality of genetic studies (Q-Genie) tool developed by Sohani et al. (17). Extracted studies received scoring from the low-quality study, ≤ 32; moderate quality study, >32 and ≤ 40; and high-quality study, >40. The risk of bias for prevalence studies was assessed using a tool modified from the guidelines in Hoy et al. (18) and Wijnenet al. (19). MFM assessed the quality of each study. Both MFM and JEA discussed the assessment results and any discrepancy was resolved by consensus.

### Data Extraction

MFM and JEA extracted the following information from each study: study aims, study population characteristics, analytical methods for the economic evaluation of IFs, types of economic evaluation, overall findings, cost results, and study conclusion. MFM and JEA discussed the extracted data and resolved any discrepancies by consensus. AW and JdV were consulted in case a decision was not reached.

### Data Synthesis

Since the included studies differed significantly in study settings, research populations, and outcome measures, we used a narrative synthesis to present details using the four types of economic evaluation techniques (cost analysis, cost-effectiveness, cost-utility, and cost-benefit) and discuss them in turn.

## Results

### Study Selection and Description of Studies

**Figure 1:**
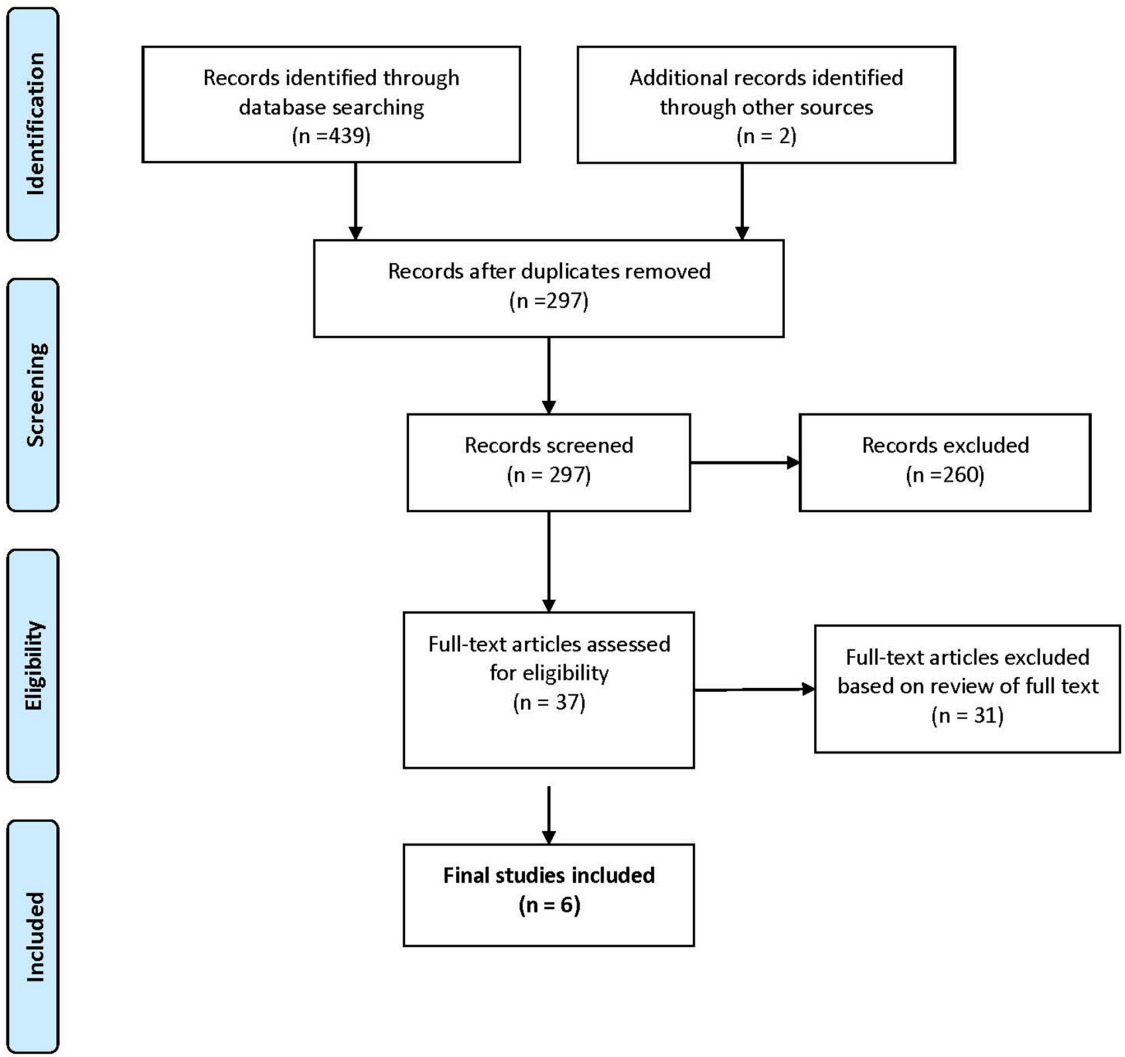
(PRISMA) diagram of results of search and selection of studies.

The search strategy for the identification and classification of articles, presented in Figure 1, shows that a total of 441 potentially eligible studies were identified that met the inclusion criteria. Following a process of reviewing the titles and abstracts, removing duplicates and articles that were not relevant (see Figure 1), 37 potentially eligible articles were subject to further analysis. Of the 37 potential articles, 31 were eliminated, 23 articles did not conduct an economic evaluation on IFs; one was a letter to the editor, one was a protocol, two were systematic reviews, and four were commentaries without empirical data on IFs. In total, six articles met the inclusion criteria (Table 1) (20–25).

**Table 1:** Characteristics of studies that met inclusion criteria.

**References**	**Study design**	**Research populations**	**Type of economic evaluation**	**Provide cost results?**	**Overall cost for return of IFs**
Bennette et al. (20)	Cross-sectional	Cardiomyopathy patients, colorectal cancer patients, or healthy individuals	Cost analysis	Yes	Returning IFs was expected to increase lifetime health-care costs by ~$90 on average for each cardiomyopathy patient, $325 for each colorectal cancer patient, and $440 for each generally healthy individual who received genomic sequencing
			Cost-effectiveness		Patients were expected to gain between 0.25 and 0.57 QALYs on average after returning pathogenic variants, depending on their underlying clinical condition, as compared with carriers whose pathogenic variants remained unknown.
Christensen et al. (21)	Randomized controlled trial	100 cardiology patients with cardiomyopathy diagnoses, and 100 ostensibly healthy primary care patients	Cost analysis	Yes	Cost reduction of omitting or limiting the types of secondary findings was < $69 and 182 per patient in cardiology and primary care, respectively
Hart et al. (22)	Cross-sectional and semi-structured interview	CSER projects participants	Cost analysis	Yes	ACMG IFs average cost up to a 1-year period was $421
Marshall et al. (23)	Cross-sectional (online survey)	General population sample of adults (21 years and older)	Cost-benefit	Yes	62% would pay for IFs. Average willingness-to-pay estimate was $299 for the basic report and $180 for a medical treatment which is currently unclear
Regier et al. (24)	Discrete choice experiment (DCE)	General population sample of 18 years of age or older	Cost-benefit	Yes	Average willingness to pay was $445 to receive IFs in a scenario where clinicians returned information about high-penetrance, medically treatable disorders.
			Cost-utility		Most participants valued receiving IFs, but personal utility depended on the type of finding, and not all participants wanted to receive incidental results, regardless of the potential health implications
Valencia et al. (25)	Longitudinal	Forty pediatric patients	Cost analysis and Cost-effectiveness	No	–

*A study may have addressed more than one type of economic evaluation in its analysis. ACMG, American College of Medical Genetics and Genomics; QALY, quality-adjusted life-year; DCE, discrete choice experiment; WTP, willingness to pay; ASD, Autism Spectrum Disorder; CSER, Clinical Sequencing Exploratory Research; IFs, incidental findings*.

In summary, all the studies included in this review were of high to moderate quality, with a low to moderate risk of bias. We reported the main results in terms of Drummond et al. (14) type of economic evaluation used and the overall cost of returning IFs to individuals. Table 1 shows the overall results of different economic evaluation types. Out of the six studies included in this review, the most common economic evaluation types were cost analysis (*N* = 4) and cost-benefit (*N* = 2), followed by cost-effectiveness (*N* = 2) and cost-utility (*N* = 1) (note that a study may have addressed more than one type of economic evaluation in its analysis).

### Cost Analysis Results

One out of four cost-analysis studies (25%) did not provide cost results, the study provided only the number of other genetic tests done per patient prior to the WES testing (25). For the studies which provided monetary values, returning IFs to patients would cost on average a lifetime healthcare cost of ~$90 for a cardiomyopathy patient, $325 for a colorectal cancer patient, and $440 for a healthy individual (20). While Hart et al. (22) found that returning IFs up to 1-year would cost on average $421 per patient. However, Christensen et al. (21) found that a patient in cardiology and primary care would save ~$69 and 182, respectively, by omitting or limiting the types of IFs returned.

### Cost-Benefit Results

Cost-benefit studies used the willingness to pay approach to assess the cost of IFs results. One out of the two cost-benefit analysis studies (50%) provided no cost-benefit results, Marshall et al. (23) study found that patients are willing to pay on average $299 for a basic genetics report and $180 for results that might not provide a clear medical treatment. While Regier et al. (24) suggested that patients are willing to pay on average $445 for actionable results.

### Cost-Effectiveness Results

One out of the two cost-effectiveness analysis studies (50%) had not provided statistical results (20, 25). The study reported that WES testing was more cost-effective than other genetic tests; however, its conclusion was based on descriptive statistics considering the number and the types of genetic tests done by the patients before the WES testing and not on the difference in the monetary values or health outcomes gained between genetic tests (25). The only study that provided findings on the cost-effectiveness of IFs suggested that patients who receive pathogenic variants test results are expected to gain on average between 0.25 and 0.57 QALYs (20). The study concluded that the cost-effectiveness of returning IFs (e.g., cost per QALY gained) was mainly dependent on the patient underlining conditions and the number of pathogenic variants returned.

### Cost-Utility Results

Only one study on cost-utility analysis provided cost-utility results. Regier et al. (24) found that 76% of the participants value having a choice about what type of IFs they would receive. However, personal utility depends on the types of finding (e.g., high-penetrance, treatable disorders, or receipt of information about high penetrance disorders with or without available treatment), and not all participants wanted to receive incidental results.

## Discussion

While there is a vast discussion on how IFs should be managed and the ethics behind the delivery of its results (26, 27), this study shows that literature on the economic evaluation of returning IFs in genomic sequencing is very limited. This formal economic evaluation will likely inform policies decision and actions to take, particularly in settings, such as Africa, that have not yet clearly defined guidelines on how to handle IFs in genetic medicine practice and genomic research. We found six published articles that met the eligibility criteria of this study with some articles using more than one type of economic evaluation. Two articles used cost analysis only, two used cost-benefit analysis only, two used both cost analysis and cost-effectiveness, and one used both cost-benefit analysis and cost-utility to describe the cost of returning IFs in genomic sequencing.

These findings indicate the paucity of data on the economic evaluation of returning IFs in genetic research. Although 71% of the studies in this analysis provided some sort of economic evaluation results (five out of seven), these results do not provide sufficient evidence to understand the cost and benefits of returning IFs to patients fully. Similar to the results of this study, a scoping review considering the cost-effectiveness of clinically actionable findings with WGS found that only 29% (seven out of 24) of studies addressed IF conditions (15).

Among the studies which provide economic evaluation results, our findings suggest that the cost of returning IFs depends on the primary health condition of the individual. For instance, Christensen et al. (28) found that the lifetime average health care cost of returning IFs for a cardiomyopathy patient is around $90, compared with $325 for a colorectal cancer patient, and $440 for each healthy individual. Christensen et al. (21) results show that omitting or limiting the types of IFs results that patients in cardiology and primary care receive can save on average $69 and 182, respectively. Hart et al. (22) add to the discussion that returning IFs to patients will increase healthcare resource utilization and the cost on average is $421 (range $141–1,114) up to 1-year post-result return of the IFs.

Although we have limited information on the exact cost of returning IFs results to the patient, there is an increasing concern in the literature that returning IFs to patients might not be worthwhile to pursue as IFs results may provide limited clinical benefits to individuals. The results of the study show that there is limited evidence of the cost-effectiveness of returning IFs to the patients. Similar to these results, Hart et al. (22) suggest that there is not enough data to fully evaluate the benefits, risks, and costs of returning IFs to patients. On the other hand, Bennette et al. (20) found that returning pathogenic variants, depending on their underlying clinical condition, might be cost-effective as patients may gain on average between 0.25 and 0.57 QALYs.

However, it is not clear that returning IFs might be cost-effective, the cost-benefit and cost-utility results show that patients included in the studies we reviewed, indicated a willingness to pay for IFs results. They also indicated wanting to have a choice of what type of findings they would receive (24, 29). It is not clear whether patients in African genomics studies would equally be willing to pay to receive IF results. Hart et al. (22) findings on experiences of participants receiving IFs, show that although there were mixed psychological reactions from receiving IF results, such as ignoring the results to feeling scared and trying to avoid thinking about the results, participants in that study did not regret opting for receiving IF results. Returning IF results to patients without proper diagnostic information may lead to a “diagnostic misconception” (11) and cause negative effects, such as false disease diagnosis, psychological distress, and financial burden.

Although the few studies reviewed point to some nuances in the cost of returning IFs to patients and health service users, there is still a need for more analyses for an in-depth understanding of the costs and benefits of returning IFs, including studies particularly situated in African healthcare contexts. For example, what are the characteristics of the patients who are more willing to pay for returning IFs results? What are the characteristics of the patients or health service users who will prefer not to receive IFs results? The increasing recognition of the contribution of genomic research to disease prevention provides fertile grounds for countries to invest in prevention. However, this needs to be balanced with competing demands for limited resources, especially in lower and middle-income countries. If there is a strong desire to return IFs to patients, more scientific knowledge on the costs vs. the benefits of returning IFs will be useful for policy negotiations to, among other things, make them affordable to health service users who need them.

## Limitations

One of the research limitations is that the systematic review only considered three databases (Medline through Scopus, PubMed, and Google Scholar); yet, these databases are popular and widely used for genetic research, and a reference list search was performed. Another limitation is that we only included studies conducted in English. Some other studies that may meet the inclusion criteria but written in other languages were not included in the analysis. Also, due to the limited amount of research on the topic, our conclusion on the cost/benefits of returning IFs cannot be extrapolated to the general population.

## Conclusion

This study results show that there is a cost associated with returning IFs to patients. However, this cost depends on the primary health condition of the patient. Although the cost may be high, many patients are willing to pay for returning the IF results. In general, patients place value on the IFs results and want to have a say on what type of results should be returned to them. This review calls for additional research to understand the cost and benefits of returning IFs to patients fully. Future studies or programs aiming at returning information regarding IFs to patients should first evaluate the adverse cost/benefits of returning IFs before returning IFs to patients.

## Author Contributions

MFM and JEA: conceptualization and methodology. MFM: data curation, formal analysis, writing, and original draft. JEA, JdV, and AW: supervision and writing, review, and editing. All authors contributed to the article and approved the submitted version.

## Conflict of Interest

The authors declare that the research was conducted in the absence of any commercial or financial relationships that could be construed as a potential conflict of interest.
